# Becoming a first-time father during the COVID-19 pandemic in France

**DOI:** 10.3389/fpsyt.2024.1376934

**Published:** 2024-04-16

**Authors:** Romuald Jean-Dit-Pannel, Rose-Angélique Belot, Denis Mellier, Laura Robert, Célia Petersen, Benoît Dinet, Cécile Bréhat, Flora Koliouli

**Affiliations:** ^1^ Psychology Department, Laboratory of Psychology, Faculty of Languages and Human Sciences, University of Strasbourg, Besançon, France; ^2^ Department of Family Medicine, Laboratory of Psychology, Faculty of Health, University of Franche-Comté, Besançon, France; ^3^ School of Early Childhood Education, Faculty of Education, Laboratory Psyché, University of Thessaloniki, Thessaloniki, Greece

**Keywords:** first-time father, perinatal, COVID-19, subjective experiences, depressive symptoms, projective tests

## Abstract

The role of fathers in Western societies has undergone significant change over time. However, their psychopathology remains largely misunderstood and difficult to identify. This study aims to explore the lived experiences of first-time fathers during the COVID-19 pandemic. Twenty-seven first-time French fathers were recruited for the study, which involved a narrative interview, the Rorschach projective test, the Edinburgh Postnatal Depression Scale (EPDS), and a semi-structured interview. Narrative interviews revealed several challenges faced by these fathers, including the pressure of paternal responsibility, the need to be actively involved in the prenatal activities and caregiving (haptonomy, skin-to-skin contact), and concerns about the future of their couple and family as a triad. The Rorschach tests showed numerous perinatal responses and difficulties in identifying phallic representations among the fathers. Additionally, the EPDS scores indicated that 15% of fathers showed signs of depression, while 52% exhibited signs of anxiety. This study also examines the impact of the COVID-19 pandemic and its associated health context on creating the initial triad. Lastly, the case of one father is presented to illustrate the need for diagnostic tools to address the psychopathology of fathers, as narrative or semi-structured interviews have often fallen short of addressing this issue.

## Introduction

The transition to fatherhood is a challenging period in man’s life. It is crucial to consider social factors, including hegemonic masculinity and paternal involvement, when assessing contemporary fatherhood (1). Although masculinities and paternal roles have changed significantly since the 1970s and the 1980s (2–4), there is still a limited understanding of the psychopathology of men. Psychopathological events are often separated from the perinatal period by healthcare professionals (5, 6) and are not easily distinguishable from the range of emotions experienced by men during this time, including depression and depressivity (common among men during this period) (7).

Perinatal mental health has drawn attention in recent literature because of the high prevalence and impact of anxiety symptoms and disorders during the perinatal period (8, 9). Many studies have investigated perinatal mental health in terms of stress, depression, and postpartum depression from the mothers’ perspective (10–12). However, most studies have been conducted using self-report scales. In a recent study conducted by Belot et al. (13), researchers utilized the Rorschach test to gain insight into the psychological adjustments that occur during the postpartum period in mothers. As stated by (13, p. 74), this method enables researchers to observe the unique aspects of an individual’s internal world and the intensity of their projective movements. The Rorschach test is particularly useful for updating an individual’s internal reality, including their body image and self-representation, as well as their representation of early relationships.

Despite the attention drawn on the specific features of Rorschach during the perinatal period among mothers (14–16), the father’s perspective remains unexplored (17–19). However, at the onset of fatherhood, they may experience neurotic (perinatal paternal depression), psychosomatic (broodiness, couvade syndrome, lower testosterone levels, and higher progesterone levels), and psychotic (postpartum psychosis) manifestations (20, 21). These issues should be considered in light of the various stages of the transition to parenthood: conception, announcement of pregnancy, ultrasound scans, childbirth, initial bonding, back-to-work period, and first caregiving arrangements (day-care center, childcare assistant, school) (22, 23). These phases are very sensitive (vulnerable) for first-time fathers. According to existing literature, first-time fathers’ needs correspond to specific professionals supports, preparation and guidance before childbirth (24). These fathers need to schedule appointments with healthcare professionals for both antenatal and postnatal services to discuss their emotional and psychological transition (25).

During the prenatal period, COVID-19 has further disrupted their presence and active involvement. In a recent qualitative study (26) exploring paternal posts online, it seems that the pandemic-related uncertainty about COVID-19 has resulted in increased self-reported fears and anxieties during the perinatal period, while fathers have also expressed the negative impact of social isolation on the transition into parenthood, which often relies heavily on the support of family and friends.

First-time fathers’ mental health and wellbeing is, in every case, an emotional roller coaster (27, 28). Some men may resort to coping mechanisms such as denial or avoidance (e.g., engaging in sexual activities outside of their relationship, avoiding responsibilities, smoking, working longer hours, or listening to music) (29). Additionally, fathers may experience specific fears related to labour and birth processes (30–32). Moreover, paternal mental health in terms of anxiety and depression may have an impact on the paternal involvement and parenting style (32–34).

Persistent gender roles about parenthood imply (35) that men continue to identify mothers as the primary caregivers and sole parents. For fathers, work schedules and traditional beliefs about breastfeeding can hinder their involvement. Despite this mindset, research reveals that when fathers invest more time with their infants, they can identify with the mother’s caregiving abilities (36). The COVID-19 pandemic has also impacted paternal involvement in caregiving, contributing to gender equality (37). Studies indicate that fathers have experienced positive consequences of remote work and furlough, as well as increased work-related stress (38, 39). As a result, the pandemic’s impact on work schedules has led to shifts in household tasks and childcare responsibilities. Research from Italy suggests that fathers’ increased involvement can enhance children’s emotional well-being (40), as they take on more domestic tasks. In Germany, fathers with lower or medium education levels spent more time with their children during the first lockdown than ever before (41).

In France, fathers experienced the lockdown as an opportunity to be more available to their toddler’s needs and rhythm (42). Men’s presence at home differed depending on their on-site or telecommuting professional activities, with unequal amounts of time off taken. Nevertheless, in the context of perinatal care, restrictions on access to prenatal care, such as ultrasound scans and childbirth classes were set for fathers. More specifically, during the first lockdown (17 March to 11 May 2020), pregnancy monitoring has been largely impacted. According to Doncarli et al. (43), 41% had an unsuccessful attempts to exchange with health professionals about the course of pregnancy/childbirth during pandemic; 39,4% had teleconsultations by video or telephone for pregnancy monitoring; 91.8% experienced an absence of partner/person providing support from a consultation/examination; 15,2% had childbirth preparation sessions by video or telephone; 36.3% had a modification of pregnancy monitoring at the initiative of a health professional and 23.4% at the initiative of the women) and less premature childbirths has been observed (44). Furthermore, 18 0745 childbirths between March and April have been inventoried and 11 7581 childbirths in November and December (second lockdown was from the 30 October to 15 December 2020), either globally 73 5196 for 2020 (https://www.insee.fr/fr/statistiques/serie/001641601). Less childbirth (-10,2%) was observed from 15 December 2020 to 15 February 2021 (comparatively to the same period one year before, from 15 December 2019 to the 15 February 2020) due to the first lockdown. A bounce has been observed in March-April 2021, then nine months after the second lockdown (https://www.insee.fr/fr/statistiques/5427433) until the end of 2021. Nine months after the third lockdown (from 3 April to 3 May 2021), we observed in France less childbirths (-2,9%), then another bounce in February 2022.

However, little is known about their subjective experiences during the COVID-19 pandemic in a perinatal context. The aim of this paper is to explore and analyze this first-time fatherhood experience during the pandemic and highlight the latent psychopathological risks.

## Method

### Research design

To capture the subjective experiences of first-time fathers during the pandemic, we used an experiential approach (45) to gather rich and meaningful data, which were effectively informed by a psychoanalytic perspective (46). The experiential approach involves encouraging participants to reflect on their personal experiences and emotions as well as the impact of the pandemic on their lives. Additionally, incorporating a psychoanalytic perspective can provide insights into unconscious thoughts and feelings that may influence their experiences.

### Participants

French speaking fathers over 18 years old whose newborns were aged between zero and eight months were included in the study. Moreover, both parents had to be first timers to be considered. Exclusion criteria included fathers whose partners gave birth prematurely, or who had obstetric complications or somatic illness during pregnancy and delivery. Moreover, complex pregnancy and childbirth situations, such as infant resuscitation, were also excluded. Single fathers, adopting or foster parents with a diagnosed psychiatric pathology were not included in this study.

Thus, 27 first-time fathers were enrolled in this study after the birth of their children (irrespective of the maternity ward in which they were born), using interviews conducted at home between October and November 2020. The recruitment process was carried out by psychology students from the University of Franche-Comté in Besançon and a medical professor who is also a practicing physician at the university’s medical faculty. Approximately one out of four fathers agreed to participate in the study.

### Data collection

Data collection occurred in the premises of participant’s i.e. their homes. Men becoming fathers answered to the following protocol: (1) narrative interviews (47–49), (2) the Rorschach projective test (50–52), (3) EPDS (53–57), and (4) semi-structured interviews. The purpose of using two different types of interviews (that were conducted during the same session) occurs from the different type of data we wanted to achieve. For instance, a narrative interview is a type of interview where the interviewer allows the interviewee to tell their story in their own words. The interviewer provides minimal prompts or questions to guide the conversation, and the interviewee is free to share their experiences, thoughts, and feelings (45, 48). The minimal interventions allow the interviewee to reflect about what they were saying, and the interviewer remains silent. According to Fontanella et al. (48), “the interviewee’s silence does not necessarily mean a conclusion of his/her reasoning, an inhibition or a disinterest, but it has many *psychological meanings* to be interpreted, such as the search for the best form to elaborate mentally what he/she feels and imagines for example” (48, p.815). On the other hand, a semi-structured interview is an interview where the interviewer has a pre-determined set of questions or prompts to guide the conversation. The interviewer may have some flexibility in asking follow-up questions or probing deeper into certain topics, but the overall structure of the interview is predetermined, in our case on COVID-19 experiences.

### Narrative interviews

The narrative interviews began with a simple question, designed to be as broad as possible to encourage a discussion which is more than an introduction to the subject: “Would you like to tell me about your feelings, your experience of becoming a father?” We deliberately chose to stress the experience of becoming a father before invoking the COVID-19 pandemic. Fathers rarely spontaneously mentioned this latter issue at the beginning of their wording.

Following the non-directive nature of the narrative interviews, we respected the silences of the participants, and the prompt questions were used to further develop their narratives (i.e. “Could you please further explain? Could you give us an example? “What do you mean by…?”. Thus, the narrative interviews lasted from 2 to 29 minutes, 11 minutes on average. It seemed that some fathers felt unease with the silences that are sustained during the narrative interviews, thus few narrative interviews were short in duration.

### The Rorschach Inkblot Test

The Rorschach Inkblot Test (RIBT) is a standardized projective technique (50, 51). The main purpose of the Rorschach Inkblot Test is to assess personality functioning. It explores defense mechanisms, emotional functioning, and thinking patterns with ten Blot cards. This tool can be used as a therapeutic mediation by the way this test is offering a unique occasion to project answers linked to psychic functioning (in a conscious and in an unconscious way). Also, “because of its nonfigurative nature, which is structured around an axis” the Rorschach Inkblot Test “gives indications of the participants’ body image and the quality of their identity and narcissistic construction” (58, p. 11). We focused particularly on the following cards:

- Blot card IV, according to (59, p. 108), evokes images that symbolize power;
- Blot card VI, with its strong sexual symbolism (59), represents a phallic or penial dimension;
- Blot card VII, as described by (59, p. 109), features white spaces in the center that may represent emptiness, a sense of lack, and challenge the inner and outer worlds, potentially evoking the mother’s womb;
- Blot card IX, as suggested by (59, p. 110), may evoke representations of the body’s interior, with its disruption of inner-outer limits or transparency of the envelope, revealing uncovered organs and viscera; and finally,
- Blot card X, the last one, represents separation, a central issue in perinatal care, with its scattered nature testing the unification of the body image (59).

### Edinburgh Postnatal Depression Scale

The Edinburgh Postnatal Depression Scale (EPDS) originally developed by Cox et al. (60), contains 10 items with four response options each rated 0–3. EPDS is a self-administered screening tool to evaluate postpartum depression. Thus, the range is 0–30 points, where higher scores indicate more depressive symptoms. The EPDS asks if, during the last seven days, the respondent had been able to laugh/see the funny side of things, looked forward to things with enjoyment, blamed oneself unnecessarily when things went wrong, been anxious or worried for no good reason, felt scared or panicky for no good reason, felt that things had been “getting on top” [of the respondent], had been so unhappy that it has been difficult to sleep, had felt sad or miserable, had been so unhappy that [the respondent] has been crying, and if the thought of harming oneself had occurred to the respondent. The EPDS has also been validated for fathers by Matthey et al. (57), who demonstrated that score 9/10 or more was optimal for detection of minor and/or major depression, with a sensitivity of 71.4%, a specificity of 93.8%, and a positive predictive value of 29.4% (61). We used the French validation by Guedeney and Fermanian (54) and (44).

We tried to be very vigilant when participants obtained high scores on the following items: item 10 (related to suicidal ideation: “I have occasionally thought of harming myself”), item 3 (“I blamed myself, for no good reason, as being responsible when things went wrong”), item 4 (“I felt worried or concerned for no reason”) and item 6 (“I’ve had a tendency to feel overwhelmed by events”). In these cases, we proposed further diagnostic and care and referred to a professional, notably when item 10 was very high scored.

### Semi-structured interviews

The purpose of using semi-structured interviews was to delve further into certain aspects of their experiences with COVID-19 as parents. Specifically, we aimed to evaluate their perceptions of the impact of COVID-19 and lockdown on their lives. During the second phase of the interview, thus the semi-structured interview, we asked each participant seven group of questions as follows: we inquired them about (1) their COVID-19 status, i.e. to share their experiences about the COVID-19 impact (whether they have contracted the virus, how did they experience the general context and sanitary restrictions as per their experience of becoming a father); (2) whether they were able to attend prenatal examinations with their spouse; (3) whether they were present during labor and whether they were wearing masks during the delivery; (4) whether they could practice skin-to-skin contact with their newborn after the delivery, and we asked them to share their experiences and feelings about it; (5) whether they cut the cut the umbilical cord (in French NICU’s is a common practice for fathers to cut the cordon and engage to skin-to-skin contact). Finally, we asked them about (6) the duration of their stay at maternity ward (in days) and (7) whether they were allowed to visit their newborn (yes/no). The mean duration of the semi-structured interviews was 17 minutes (*from 5 to 25 minutes*).

### Data analysis

For the narrative interviews, we used NVivo 12 and followed narrative thematic analysis (45). More specifically, we developed the initial themes that emerged from the data themselves. These themes were based on the experiences and perceptions of the participants in our study. Then, after identifying initial themes, we have refined them by checking (reviewing) data again and looking for additional evidence to support our themes. At this point, some themes were merged, and others were more developed. Finally, we wrote up analysis. To ensure the validity of our analysis, we got feedback from the research team. For the semi-structured interviews, we proceeded in the same way: i.e. we have generated codes that occurred from the data. Then, we tried to organize these codes into broader themes and so on. Moreover, prompt questions were sometimes close-ended, which helped us with the analyses.

The Rorschach Inkblot Test results were analyzed with the Comprehensive System and the French school method (52, 58, 59, 62, 63). Each response to the Rorschach cards was scored according to the criteria and technical standards of the Parisian School (59), as it has strong psychometric indicators in France (64). Trained researchers administered the test.

Thus, we followed the French School analysis according to which “has been consistent in this methodological interpretation of the Rorschach that values a system of coding, comparing the collected data with general population norms, and developing clinical hypotheses that are put into practice through a rigorously codified qualitative analysis, articulated together with the analysis of the protocol (58, p. 9)”. The EPDS analyses (55) were considered with French literature (53, 54, 57).

### Ethical considerations

The study was conducted in accordance with the local legislation and institutional requirements (Department of Psychology of the University of Franche-Comté, in France). The participants provided their written informed consent to participate in this study, before the data collection procedure started. Due to the constraints posed by the ongoing pandemic, the researchers have secured digital letters of permission with affixed e-signatures from the participants. The participants were additionally asked verbally if they consented to be interviewed during the scheduled date of their interviews. Only participants who were 18 years or older at the time of their consent were interviewed for this research. Participants were encouraged to narrate their experiences freely and honestly in response to the given questions. They were also given the discretion to refuse to answer a question or cease participating anytime if they wished to do so. Furthermore, considering the sensitive nature of this research, participants were allowed to contact a trained psychologist/therapist after the interview if needed. An informed consent for recording the interview was also given by the participants who were assured of the humane treatment and strict compliance to the confidentiality of personal information throughout the whole research.

## Results

In this part, we will present the narrative thematic analysis of both interview types of interviews, followed by the Rorschach analysis and the EPDS (see **Table 1**, **2**). The data collection procedure lasted between 30 minutes to one hour. We will finally present a case study (father 24) because his EPDS score was high (showing severe mood dysregulation) and had several shock reactions at Rorschach test despite interviews where everything seemed to be fine for him.

**Table 1:** Sociodemographic data of N = 27 fathers.

Father	1	2	3	4	5	6	7	8	9	10	11	12	13	14	15	16	17	18	19	20	21	22	23	24	25	26	27
**Child gender**	M	F	M	F	F	F	F	M	F	M	M	M	M	M	F	F	F	F	M	F	M	M	M	M	M	F	F
**Month of birth (2020)**	05	09	05	04	04	07	04	04	11	08	04	05	08	08	10	07	05	07	05	07	06	09	05	07	07	05	06
**Child’s age (at the time of the interview) (m,d)**	6 m	1 m 23 d	5 m 27 d	6 m 18 d	2 m	4 m	7 m 3 d	6 m	6 d	3 m 17 d	7 m 29 d	3 m	4 m	3 m 3 d	17 j	4 m	5 m	4 m 15 d	5 m 15 d	4 m 15 d	5 m 15 d	2 m	5 m 15 d	5 m 1 d	4 m	6 m	5 m
**Father’s age**	28	34	31	31	30	26	31	26	28	28	40	24	33	30	36	35	30	31	29	38	39	31	30	34	28	33	26
**Father’s SPC**	3	3	5	3	3	3	4	5	3	5	3	6	5	5	5	6	5	4	5	4	6	6	4	5	3	3	6
**Mother’s age**	27	31	30	30	28	26	30	25	27	25	36	26	31	30	35	30	30	29	31	32	30	34	29	32	30	32	25
**Mother’s SPC**	3	3	3	4	4	3	3	3	4	3	3	5	4	SE	5	FF	5	4	5	4	5	SE	4	4	4	4	5

SE, unemployed; FF, housewife.

**Table 2:** Traumatic events during delivery.

Father	1	2	3	8	13	14	15	18	19	24
**Traumatic delivery**	Baby had an operation	Blocked placenta	Baby got his shoulder stuck	Hemorrhage	Cord around the neck	Dehydration	Tear	Caesarean section, resuscitation	Risk of infection	Epidural, tear

### Socio-demographic description of the final sample

The fathers in our study (see **Table 3**) were *M* = 31 years old (aged between 24 and 40), of the following socio-professional categories (French SPC)^1^: Executive or technical staff or intellectual profession (SPC3 = 9), intermediate profession (SPC4 = 4), employees (SPC5 = 9), and workers (SPC6 = 5). The average age of their infants was four months and nine days (between six days and seven months 29 days), and the gender distribution was nearly equal, with an almost equal number of girls and boys. The average age of the mothers was 29 years (range, 25–36 years), and they mostly belonged to the following socio-professional categories: Executive or technical staff or intellectual profession (SPC3 = 8), intermediate profession (SPC4 = 10), and employees (SPC5 = 6). A total of two mothers were unemployed and one was described as a housewife (SPC6 = 3). It is worth mentioning that 3 of pregnancies were declared unplanned by these fathers (fathers 16, 23 and 26), even though they had been previously envisaged by the couple. Finally, only one father mentioned having contracted the COVID-19 virus (father 13). All fathers were presents during the delivery. Overall, there was no perceived economic impact on their professional activity, which is important to mention in this financially sensitive perinatal period.

**Table 3:** Results on close-ended questions of semistructured interviews and tests.

Father	1	2	3	4	5	6	7	8	9	10	11	12	13	14	15	16	17	18	19	20	21	22	23	24	25	26	27
**Number of answers to Rorschach**	13	14	16	15	9	9	14	18	14	18	15	11	10	14	11	13	12	23	13	14	21	15	20	14	30	10	11
**Shock (+20 secs)**	6, 9	7, 8	N	N	4, 6, 7, 8	7	6, 7	6,7,9	N	N	2	5, 4	7	4, 6	6, 4, 9, 10	6	6, 9	9	2,4,7,9	6,10	N	8	N	1, 4, 6, 10	NK	NK	NK
**Cards +**	8	2	8, 3	6, 8, 4, 2	3, 9, 10	1	10	N	2	10	10, 1, 3	7	10	5, 6	8, 5	10, 9	7	9, 7, 10	1	10	3, 7, 9	6	3,10	7	10, 9, 8	NK	1
**Cards -**	2	4	7, 6	10	4, 7	10	4	4, 6	8, 10	4	4, 6	4	2, 4	4, 7	2	1, 4	4, 6	6, 4, 5	9	2	5	8	1,4,6	2	4	NK	6
**EPDS (Score)**	2	7	**12**	9	8	2	**12**	6	7	2	1	1	7 (2 at 10)	6	6	9	10	1	7	4	5	5	4	17	2	7	6
**2 Absence of the father at maternity (all long, day and night)**	Y	Y	Y	Y	Y	Y	N	Y	Y	Y	Y	N	Y	Y	Y	Y	Y	Y	Y	Y	Y	Y	Y	Y	Y	Y	Y
**Time at home (professional activity)**	3w	4w	3w	3w	4w	6w	4m	2w	NK	2w	14w	3w	2w	2w	2w	4w	12w	2w then wah	Cpl w	NK	3w	3w	4m	7w	NK	NK	NK
**Planned pregnancy**	Y	Y	Y	Y	Y	Y	Y	Y	Y	Y	Y	Y	Y	Y	Y	N	Y	Y	Y	Y	Y	Y	N	Y	Y	N	Y
9 unable to attend **ultrasounds**	N	Y	Y	Y	N	Y	N	N	N	Y	Y	Y	N	N	Y	Y	Y	N	Y	N	Y	Y	Y	Y	Y	Y	Y
**18 wore masks during the delivery**	Y	Y	N	N	Y	Y	N	N	Y	N	N	Y	Y	Y	Y	Y	Y	N	Y	Y	Y	Y	NK	N	Y	Y	Y
**20 praticed skin-to-skin**	N	Y	N	Y	Y	Y	Y	Y	Y	Y	N	Y	Y	Y	Y	Y	Y	Y	Y	N	N	N	Y	Y	N	Y	Y
**3,80 days at the maternity**	5	5	3	4	3	NK	3	5	5	2	6	2	3	7	3	1	3	5	5	5	3	3	3	4	2	6	3
**6 had external visits at the maternity unit (family and friends)**	Y	Y	N	N	N	N	N	N	N	Y	N	N	N	Y	Y	N	N	N	N	Y	N	N	N	N	N	N	N
**20 fathers cut the cord**	Y	N	Y	Y	Y	N	Y	Y	N	Y	Y	Y	N	Y	Y	Y	Y	N	Y	Y	Y	Y	Y	Y	N	Y	N

Nk, Not know; W, week; M, month.

### Phase 1: narrative thematic analysis

The following analysis occurred from the convergent data analysis from both types of interviews. It explores first-time fathers’ experiences of becoming a father during through the examination of two superordinate themes. The first one is named “Becoming a first-time father,” the second one “Lockdowns, COVID-19 and access to fatherhood.” The first theme describes the shift in emotional states that participants experienced and is distinguished in subthemes i.e contrasting emotions and emotional investment, “perfect timing-s”, “shifting from dyad to triad”, and “extended family presence”. The second one includes the experienced uncertainties associated with the lockdown and the COVID-19 pandemic associated with the promotion of paternal involvement. For ease of explanation, the two themes were presented separately, although they are associated.

### Becoming a father for the first time

#### Subtheme 1. Contrasting emotions and emotional investment

In our study, approximately half of the participants viewed the onset of fatherhood as a joyous occasion, but it also brought about a sense of responsibility that could be both advantageous and unsettling. This feeling was connected to specific subthemes, including worry for nine fathers, anxiety for eight, and responsibility for seven.

Father 1’s narratives illustrated the experienced contrasting emotions:

“The delivery was the happiest day of my life. I began to cry when I saw him for the first time. I was instantly overjoyed, but in reality, it was much more than that [ … ] I was also allowed to attend and cut the umbilical cord. I was afraid I would not be able to do this because of the pandemic, as I hadn’t been able to attend the final ultrasounds.”

“The only downside to it all is that we had to wear masks, and I wasn’t allowed to touch my son immediately after the delivery for safety reasons.”

Fathers in our sample demonstrated their dedication and participation in a tangible manner by readying their unborn child’s room as if they were preparing themselves mentally, wallpapering or painting a nurturing environment that aligns with their pregnancy experience.

#### Subtheme 2. Perfect timing-s

Many participants (11/27) related largely to the concept of time and temporality. More specifically, fathers perceived that the period in which they had started their family was the optimal time for them in terms of professional status. In addition, they related to the concept of “perfect timing” in terms of their greater involvement in perinatal experiences during the COVID-19 pandemic. Father 2, for instance, presented the arrival of their baby, at a time desired and programmed by the couple – as follows:

“We waited for the moment that seemed most opportune, when we were the most settled, the readiest to welcome some small upheaval in our lives”.

The timing for this father could not have been better since he was married and had achieved stability in his professional life. Although this may have conflicted with an unexpected pregnancy in early 2020, during the outbreak of a pandemic, this issue was not explicitly addressed. According to Father 7, the baby was able to experience an undoubtably calm period during the lockdown.

“We do not know what it would have been if things had been different. However, given that she had been at home for a longer period due to closures and other factors, it seemed more convenient for both her and the little one. Currently, we have a small boy who is very sweet and smiles frequently. He rarely cries and does not appear to be stressed. Therefore, we cannot determine whether the lockdown had any impact not….”.

The same father was also more available: “I had a free, extended paternity leave”^2^. However, at the same time, both the father and their partner expressed apprehension about their child’s social skills development during the lockdown: “*We were somewhat afraid she’d become wild*”. The sense of timing concerning the lockdown was also pointed out by Father 27:

“It meant we didn’t have to drag her all over the place”, “it meant we didn’t stress her out, or ourselves, and we were able to find our own pace, and find her own pace”.

Moreover, when Father 23 evoked his son’s birth, we talked about his son’s arrival “in a new world”.

“It is fortunate that the lockdown has ended, as I could not tolerate *not* being able to assist my wife during labor and being forced to remain outside like a fool. I have a friend who was not allowed to visit his wife during her delivery due to sanitary reasons, which must have been a traumatic experience for him since it occurred during the critical period of the pandemic. Despite this weird year we had, my son is in good health, and I was able to witness his birth into a new world.”

Also, other participants acknowledged the importance of rhythm and waiting.

#### Subtheme 3. From a dyad to a triad

Fathers grappled with the significant changes in the dynamics of their marital relationship as they became parents while recognizing the advantages of the restrictive measures that established the family unit. The passage from Father 26 emphasized the chance for the triad to emerge over time, with all three individuals confined in the maternity ward, as a genuine phase of initiation into parenthood.

“I spent two or three days in the maternity ward, during which the midwives taught me various things about caring for my newborn baby, as my wife was not in the best condition after giving birth. [ … ] They even showed me how to administer first aid to my baby and advised me on the small but crucial tasks that were essential in the early days of a baby’s life. [ … ]This knowledge helped me feel more confident and prepared when I left the hospital, which was very comfortable for me….”

This passage highlights the critical role played by the father in the maternity ward after the birth of a child. By remaining present and providing support, the father allowed his partner to recover both physically and emotionally from childbirth. This care also enabled the father to take an active role as a parent, fostering a strong bond between the couple and ensuring that the mother was not alone during the early stages of motherhood.

As for Father 24, he felt that the lockdown had facilitated the development of the family nest:

“Because of the lockdown, we relocated, and we also became home-owners at the same time, so we certainly haven’t had the time to get bored”.

#### Subtheme 4. Extended family

Father 23 mentioned the significant time lapse between the birth and the baby’s presentation to the extended family, which was experienced by many families in the COVID-19 context:

“Well, it’s true that our family didn’t get to see him right away, but that wasn’t the most important thing for us! They came to see him in the weeks that followed, and we celebrated his birth in the summer [summer 2020]!”

Father 11 talked about the support and guidance that he received from his parents and parents-in-law:

“So, when she was in hospital, with COVID-19 I couldn’t stay very long. So, in the evening, at 6pm, I had to leave. It’s true that our two families, having dinner together, was a welcome relief. Since the birth was very, very stressful, we were able to discuss it with the family”.

This continued for the couple:

“Returning home was very smooth, we had nothing to do, our parents would prepare the meals, do the shopping, so we could spend time with her [their daughter] and that’s it.”

The couple were born in Spain and are originally from the same country:

“We’re from Spain and our whole family is in Spain. With the lockdown, the borders were closed so we were not sure if they could come. Especially as she’s the first granddaughter of my parents and hers”.

The parents of the first-time fathers also provided support and nurturing, reflecting certain perinatal rituals. They were therefore involved in nourishing and supporting the first-time parents, which was beneficial both to the new parents and to the newborn.

**“Lockdowns, COVID-19 and access to fatherhood”**

The second major theme of the narrative analysis englobes the experienced uncertainties associated with the lockdown and the COVID-19 pandemic associated with the promotion of paternal involvement. Father 20 described the COVID-19 related uncertainties, powerlessness, and anxiety of a man about to become a father regarding his partner and baby this way:

“My partner was still pregnant when the virus struck, and it was complicated because at first, we didn’t know what sort of virus it was. I didn’t know if my partner could catch it or even the baby. There was still no cure, and we didn’t know exactly how we would handle the situation if it got worse. It was a somewhat stressful time for us.”

Among these concerns figures the possibility to assist to perinatal examination. It appears that all the stages help to reinforce the future father’s acknowledgement of the father-fetus/baby attachment bond.

Participants stated that some experiences were decisive in their paternal involvement and investment. For instance, even though a third of fathers were unable to attend ultrasound scanning because of sanitary constraints, those who were able to attend express its importance “It began to become a bit more specific” for father 20. In the same line, haptonomy sessions were crucial for some fathers: “for me, that’s where the adventure begun” (father 2).

Regarding the issues raised by masks, we found that majority of fathers wore them in the delivery room. Some women also wore them during their delivery, but we have no accurate data regarding this. Fathers downplayed the consequences, saying, for instance, “Yes, children don’t see much at birth, so this shouldn’t be a problem” (Father 1) and “I would have liked him to see the entire me, but then I know that a newborn baby doesn’t see at birth” (Father 25).

Only two fathers, Father 7 (early April 2020) and Father 12 (late May 2020) could not be present nor stay at the maternity ward, whereas the rest of the participants could spend an average time of 3.8 days in the maternity wards. Extended family visits were possible in only a quarter of the cases. Some saw the absence of such visits as beneficial:

“Without visits, at the maternity, just the three of us, we have totally been in a bubble totally disconnected from reality. So, it was really the first feeling that really stuck me. It’s this super tight bubble around our child when she arrived. Find your rhythm, ask yourself a billion questions. (Father 2).

In the weeks following delivery, father 17 had an extraordinary experience with skin-to-skin contact that left a lasting impression on him. “*This moment is etched in my memory*” (father 17). Moreover, ten respondents reported specific events that occurred during childbirth, such as a baby needing surgery, a blocked placenta, a stuck shoulder, hemorrhage, a cord around the neck, dehydration, a cesarean section with resuscitation, a risk of infection, and lacerations among the mothers (see **Table 4**). These events caused significant trauma to the entire triad, including the baby, the mother, and the father. Father 23, however, was able to spend quality time with his baby in the French COVID-19 context and even learned to communicate with his partner using the baby language: “*it was like vacations stuck with my son, only using baby language*.”

**Table 4:** Rorschach (Father 24), R = 14, 25’.

Rorschach Card, Duration of Response, and Protocol		Scoring
**Card 1 (+)** **36’’** ➔ 1’ 1. Butterfly - 2. Beetle with front claws and wings outstretched	Insect mandibles and eyes	Criticism **Shock** /\ G F+ A Ban /\ D4/G F+ A
**Card 2 (-)** 17’’ ➔ 1’06 3. Face with open eyes and mouth (red)	Face also in white	Comment /\ D10bl F- Hd
**Card 3** 1’’ ➔ 3’’ 4. Frog	The balls above are eyes that protrude outwards	/\ G F- A
**Card 4** **45’’** ➔ 1’20 5. Monster with feet dangling and arms hanging		Symmetry comment **Shock** /\ D1 F+ H
**Card 5 (+)** 5’’ ➔ 10’’ 6. Comet moth		/\ G F+ A Ban
**Card 6 (-)** **48’’** ➔ 55’’ 7. Medieval sledgehammer	Stem = club handle, club head below. “What was used to hit each other”	Criticism **Shock** /\ G F+ Obj Comment
**Card 7 (+)** 0’’ ➔ 20’’ 8. Two people kissing 9. Two Teletubbies	Titeuf^3^; Siamese twins joined at the trunk	/\ D15 K+ H Criticism /\ Dd19 F- (H)
**Card 8** 7’’ ➔ 30’’ 10. Chameleons 11. Cortex/human body	Vertebrae, part of the anatomy	/\ D1 FC+ A Ban /\ D9 F- Anat
**Card 9** 5’’ ➔ 15’’ 12. Cow head (white) in the clouds (colors)		/\ Gbl FE- Ad Frag
**Card 10** **50’’** ➔1’20 13. Moustaches (middle green) 14. Attached wings (pink and blue)	Child with wings	Comment **Shock** /\ D10 F- Hd /\ Dd48 F- Hd Criticism

Location numbers from 59.

Some fathers also evoked the difficulties associated with their return to work. Father 2 explained that, by returning to work, he was missing important parts of the baby’s day to day live. More specifically, his “baby was sleeping in the morning” before he was “leaving to work”. In the evening, his baby “was invaded by evening anxieties”. Then he said: “out of a rainbow of emotions, I only had one color left, it was the color of anguish, the color of evening”.

The participants were able to easily discuss the COVID-19 challenges without any issues. For instance, Father 6 stated, “It seems that infants are not likely to become severely ill with the disease, which gives us peace of mind.” This helped alleviate his anxiety about contracting the virus. Additionally, Father 7 spoke extensively, using negation to express his lack of concern, saying, “I’m not worried at al “I’m not, not at all worried … not about the virus … it sounds utopian to say that, but … No, frankly, I wasn’t at all worried because … well, I said to myself, uh … I have faith in medicine. So then, she was in a room, the nurses were wearing their masks, and all that. I thought, well, if she gets sick, uh … we’ve got to stop this. So, I wasn’t worried at all, I really wasn’t, even about the virus etc … I was more scared when she came back … well, even if I was in lockdown, but I’d say I’m more scared now, because I’m still working a bit”.

### Rorschach Inkblot Test results for 27 fathers

For the purposes of this paper, we will focus on the common elements identified by the 27 first-time fathers who participated in the Rorschach Inkblot Test (see **Table 1**). On an average, they provided 15 responses each (with a norm of 28.16), as reported by de Tychey et al. (62). Analysis of their intrapsychic representations and defense mechanisms revealed a struggle within these individuals. Furthermore, some participants experienced difficulty in expressing themselves verbally, particularly when presented with blot cards that highlighted specific intrapsychic tensions. Notably, four fathers displayed up to four shock reactions (i.e., response times of more than 20 s) among the 43 shock reactions observed overall (10 shock reactions related to Blot card VI; 7 related to Blot card IV; 7 related to Blot card VII; and 7 related to Blot card IX).

The least favorite blot cards among the 14 fathers were IV and VI, which presented significant challenges in terms of both overt content and underlying demands. These cards symbolize the phallic force thwarted by the pregnancy complex and the impossibility of carrying, delivering, or breastfeeding a child. Such membership in a phallic, masculine gender may have resulted in an intrapsychic issue. On the other hand, ten fathers preferred the last card (Blot card X), which represents separation, a critical issue in perinatal care. The scattered nature of this card tests the unification of the body image. On average, respondents provided 2.33 responses, while only one response was given (between 1.15 and 1.66) for the other blot cards. Lastly, we found specific perinatal responses (uterus, vagina, baby, childbirth, ultrasound, cradle, placenta, etc.). We particularly noted 17 responses relating to the “pelvis” in these protocols. The responses demonstrate the challenges faced by first-time fathers during the perinatal period, as revealed through their projective test answers. This period had a significant impact on their identities and caused numerous internal adjustments.

### EPDS

Based on the EPDS scores, it is recommended that four out of twenty-seven fathers should consult a physician or psychiatrist for potential depression. This recommendation applies if their score is N≥10 and, depending on their response to item 10, regarding suicidal issues. The recommendation was made according to the selected thresholds, which were N≥12 or N>10.5, according to Guedeney et al. (53). Three fathers (11% of the group) fell within these thresholds.

However, the EPDS scores of the five fathers were particularly concerning. More specifically, father 17 obtained a score of 10. As for fathers 3 and 7, they scored 12, both these fathers encountered difficulties in being present to their spouses having a health problem himself (father 3) and not having access to them maternity ward (father 7). Father 13 obtained a score of 2 (“sometimes”) in response to question 10 which evokes suicidal thoughts (“I have occasionally thought of harming myself”). The participants, however, did not corroborate these suicidal tendencies. Father 24 obtained a score of 17.

Most fathers obtained higher scores on questions 3, 4, and 6:

- Fifteen fathers scored high to “I blamed myself, for no good reason, as being responsible when things went wrong” (Q3) (most of the time – 3; sometimes – 12).
- Eleven answered sometimes (8) or very often (3) to “I felt worried or concerned for no reason” (Q4).
- Finally, five fathers admitted that they ‘have had a tendency to feel overwhelmed by events” (Q6): 1 “most of the time, I felt unable to cope with situations” and 4 “didn’t feel quite as able to cope as usual.

### A case study illustration: focus on Father 24

In this section, we delve deeper into the case of Father 24. He had an EPDS score of 17 and exhibited four Rorschach shock reactions. At the time of our interaction, which lasted an hour-and-a-half, he was 34 years old and was a father to a five-month-old baby.

During the interview, he took some time to respond because of the complexity of the questions. He then rephrased this and provided a thoughtful answer. He viewed the child as a joint project between him and his partner, a long-awaited addition to their family, and everything was “ideal” to accommodate the baby’s arrival. He stated that he was not “worried,” but acknowledged that his perspective might differ from that of his partner, who actually gave birth to the child. In this sense, he saw himself more as a “companion” than a parent. He elaborated that this might explain why he was so eager for the baby’s arrival, as he fully realized the significance of the moment, he first met the baby, that is, the moment of delivery. Nonetheless, he asserted that he was well informed about what to expect and was aware of everything that was happening. He made an effort to assist his partner and do “more at home to make her feel better.”

According to this father, it was only when the child was placed in his arms, and he was informed that it was his that he fully grasped the significance of the situation. He emphasized this notion of waiting. This pregnancy was rapid for the couple, as they were already in their final weeks of the first trimester when they learned about the pregnancy. They did not anticipate the speed at which the events would unfold. The fact that they had not yet experienced the first trimester, which they considered a time filled with anxiety, may have helped them experience this phase calmly and without anxiety for the father.

When the family moved to their new home during the initial phase of the COVID-19 pandemic, they did not have time to be bored. The time spent waiting was relatively quick and comfortable for both the mother and baby. The father was joyful, referring to his child as a “nice,” “smiling,” and “unstressed” baby who sleeps well at night. He wondered whether the lockdown had anything to do with the fact that his child was so easygoing. He also stated that he was content with his job, relationship, and new home, and was pleased to see his child thriving.

After the Rorschach test (as shown in **Table 2**), this father provided only 14 responses, and he took 25 min to do so. Similarly, he struggled to respond promptly to Blot card 1. In response to Blot cards IV and VI, he displayed shock reactions such as “monster with feet dangling” and “medieval trunk” which indicate a predominantly depressive representation and stationary expression of aggression. He also faced difficulties in separating and distributing the last blot card.

The father’s EPDS score of 17, which reflects a complex mood dysregulation, could be linked to significant professional challenges. Additionally, it may have been influenced by the shortened first trimester of the pregnancy and feelings during childbirth. During these perinatal periods, he was more of an observer than an active participant. Lastly, during the semi-structured interview, he described the lockdown as an opportunity to explore and establish a family nest.

“We were discovering what it was like to have a house with a bit of a parcel of land (…) a very, very pleasant setting. Frankly, the lockdown and all that was really pleasant, and it brought us a lot of peace and quiet.” “I did as much as I could so that she didn’t necessarily have to leave the house”.

These last remarks reveal the nurturing and logistical role he played for his partner, which may have promoted fostered a paternalistic attitude towards the baby (father-baby dyad) and his partner (father-mother couple). He describes the birth of his child as follows:

“As for me, the delivery went very well… (laughs). No, were we sufficiently prepared, even if we didn’t know what lay ahead? (…) She did a good job, and yeah, I think we were well prepared.”

In this regard, he broached the subject of the umbilical cord in the following terms:

“Yes, yes, yes, but I wasn’t really given a choice. Yeah, no, I had told her ‘I don’t know if I’ll do it’ because I don’t really like all this stuff, I mean, just the thought of being vaccinated makes me want to faint, so yeah. So when he was born, they handed me the scissors and said, ‘here, you’ll need to cut here’, so you just do it and … in retrospect, it was actually a nice thing to do, but I would have asked myself a few questions if someone had told me, ‘do you want to do it’, or ‘uh, I don’t think so’. It was really because I was presented with a done deal, but it was good.”

This father expressed his experience about skin-to-skin contact in the delivery room as follows:

“She [the mother] was quite torn up, and they had to do the stitches and all, so they cleaned her [the baby], washed her up and stuff, and said to me ‘take her, go into another room…’ and I actually had skin-to-skin contact during that time. So, I had a moment, I wouldn’t say an hour but almost, you don’t see the time go by and sometimes it seems long because you’re thinking ‘oh lala but what are they doing?’ and uh … so yes, yes, I had quite a bit of skin-to-skin time. After that, I must admit that it must have been 10/11pm, we’d been awake since 4am, so we were also pretty tired.”

This father also mentioned the time spent in the maternity ward, which he described as a time for oneself and for the triad:

“In the end, the time we spent together in the maternity ward, well, it was time to ourselves. Maybe that’s a little selfish, but they were moments alone. And from what we’ve heard from the midwives, the babies born during COVID-19 have never been so calm, because not seeing so many people passing by, not being hugged, not being jostled and disturbed as they discover a world that isn’t really theirs, uh … maybe that also made it a nice moment. So, um, afterwards you obviously want to see people, we saw them three days later, but I didn’t think it was that bad, that catastrophic”.

Lastly, this father noted that the presence of masks in hospital settings “made photos … masked photos!”. Here, this new dad pointed to the veil that COVID-19 may have nevertheless placed over his memories of this perinatal period. Also, he evoked the over- and under-protection of sanitary measures as follows:

“If he’s in the stroller, we often cover him, or rather try to protect him at least, even as much as possible. But we don’t stop living though, we don’t restrict ourselves in any way. For example, after the lockdown, in August, we went out to eat. He was with us, so we didn’t completely stop living, and we didn’t say to ourselves ‘you mustn’t do that’ and so on”.

### Discussion about this first-time father

It may be difficult to see how the depression revealed by this father’s Rorschach and EPDS scores can be linked to the perinatal period, which appears “ideal” in his narrative. While his professional difficulties may help to explain this, we remain cautious in our interpretations and analysis of this clinical situation, in which psychopathological elements appear to be latent, detectable only by projective or measurement tools, given that such elements are difficult to identify through interviews.

In our opinion, several factors point to this father’s depression (65): the few responses, the four shock reactions (36, 45, 48, and 50 seconds), the absence of a qualitative color response accompanied by the presence of a single kinesthetic response (K+, card VII), and the identification of blank faces and heads.

Moreover, his EPDS score was 17. This may occur from professional or work-related challenges, potentially exacerbated by a pregnancy that was truncated to three months.

In a more general note, this father highlights the persisting representations on fathers cutting the umbilical cord for symbolic reasons (70% of fathers in our study cut the cord). However, not all fathers desire or appreciate this act, and some may fear hurting their baby or their wife after passively observing them give birth. We believe that this act should not be mandated but rather that future fathers should be allowed to question and anticipate it and change their minds at any time.

This case study generates several questions. Does this father feel the right to be exhausted by the birth of his partner and baby, and by the intrapsychic event that it represents in his life? What type of recovery is he entitled to? How could this father invest physically and psychically to his newborn when, as a father, he is still worried about his wife? The skin-to-skin practice introduces the paternal touch and tact, as it has been mentioned earlier. Although skin-to-skin is a standardized procedure in the delivery room in many French NICU’s, it seems that it may not be full experienced in its beneficial nature for both father and infant (66, 67) given that men could be extensively worried about their spouses. In this case, the father is caught between emotional fatigue and marital vigilance.

## General discussion

The aim of this study was to explore the subjective experiences of first-time fathers during the COVID-19 pandemic focusing on psychopathological risks. Salient results underline that the first-time fathers experienced the beneficial aspect of the COVID-19 and its lockdown by being present at perinatal controls, maternity and, then, at discharge in their homes. Our results are partially in line with those of existing literature. For instance, the qualitative study of Fonseca et al. (39) points out that even though fathers have missed important moments due to the pandemic such as paternal presence during prenatal controls, they also mentioned positive implications such as greater involvement in both the prenatal and postnatal periods.

First-time fathers reflected on contrasting emotions during the birth of their newborn. Current research has highlighted the contrasting or “mixed” emotions that fathers may experience at an early antenatal period, particularly at vulnerable contexts such as prematurity (67, 68). However, in the case of COVID-19, first-time fathers experienced distance and separation in terms of not being able to touch physically their child. In the psychoanalytical meaning of tactile touch (69), first-time fathers were somehow prevented from being “touched emotionally” and being able to touch their child as a form of communication and affiliation. So, how can one ensure that a triad is contained after birth (70), so that everyone is able to find their place and adapt to its rhythm (71), rather than to the rhythm imposed externally by healthcare professionals for instance, due to the sanitary context. However, fathers in our sample demonstrated their dedication and participation in a practical manner by readying their unborn child’s room that aligns with their pregnancy experience (72). In this case, the nesting process (73) was encouraged during pregnancy, considering the temporal aspect, by the lockdown situation. Furthermore, at the newborn’s arrival, fathers are involved in the caregiving process by feeding their baby, which prevented them from feeling like “dry carers” (74, 75). This expression is often used when fathers are supposed to be able to only feed their child “emotionally and psychically” (74, 75), whereas the participants of our study assumed actively their caregiving role by participating to these activities.

The fathers of our sample referred to the perfect timing of starting a family offered by the COVID-19 condition. However, in their narratives, it was not always clear whether some fathers were referring to finally meeting their baby outside the womb, to COVID-19 and its sanitary implications, or to themselves as men who had become real fathers through the process of witnessing their newborns’ birth. This consists of another novel aspect of our study. Indeed, existing research on transition to parenthood during COVID-19 (39) has not addressed this crucial dimension of timing.

In our study, another important aspect that emerges is the importance of the triadic relationship. The pandemic-specific narratives highlight the importance of considering the COVID-19 period as an initiatory time that promoted, established, and enabled the formation of an *initial triad*, which echoes the primary triad, the triadic nest (76, 77), and the primary unit of family dynamics (78). The *disquieting strangeness, the uncanny* (79), of this “new world”, for the baby and for the first-time father during the pandemic, arises here. However, given their perceptions and experiences, participants continued to view childbirth as a time of helplessness, violence, and even trauma. Some fathers also evoked the challenges associated with their return to work ending an initial continuous triadic time, a time that remains poorly elaborated within the couple and the triad, and more broadly (within the extended family, society and perinatal professionals).

Another subtheme that was developed in the participants’ narratives was the different timing in introducing their newborn to the extended family. Due to the COVID-19 context and restrictions, the discrepancies in the marking of filiations among the extended family are worth noting (80). The birth of a baby in an extended family does not always correspond to its actual date of birth, so it can be placed on hold, suspended, which introduces an asynchronized encounter, a different king of temporality, in this case.

Moreover, the sanitary conditions had exacerbated experienced anxiety related to the virus itself, (contracting the virus or transmitting it to the mother and/or newborn), and uncertainties about the health implications on the mother and newborn. In light of the psychoanalytic lens, one of our assumptions in this regard is that the ordinary primary hypochondriacal trajectory (81) was altered, i.e., a normal hypochondriacal phase in the lives of parents concerned about the health of their offspring (from embryo to toddler).

The qualitative analysis of the EPDS showed that four of the participants manifested signs of depression and that they might need to consult with perinatal professionals. Paternal perinatal depression should be distinguished that the maternal one: usually, fathers show less severe symptoms, and mood alterations are often in comorbidity with other affective disorders. Moreover, paternal perinatal depression negatively impacts on family functioning, on couples’ relationships, and on family members’ well-being (82–84 et 85).

Using the results of the Rorschach Inkblot test, we discovered the extent to which new fathers are in a unique psychic dynamic, intrapsychically intruded, and reworked. We found many anatomic responses linked to the woman/mother’s body during this perinatal phase, and the usual phallic or masculine responses were missing, especially at cards 4 and 6. The identity of these first-time fathers was profoundly disrupted, with answers such as death, skull, skin, dead crushed disemboweled or boneless animals, and dead leaves. We generally found that masculine and phallic images were impacted for these men due to their transition into fatherhood, as observed in the results for cards 4 and 6 (59). The EPDS results were associated with the Rorschach outcomes. As a result, anxiety, depression, and even major depression could be detected. Without this tool, it would not have been possible to identify these issues based on our interviews alone. Moreover, even when specific tools make it possible to identify these signs, the results remain difficult to apply and interpret in the context of both research (in our case, research) and healthcare. Consequently, our findings suggest that there may be a need to administer a simple and easy-to-use scale, such as the one presented here, to fathers during the perinatal period. Indeed, this may provide an opportunity to identify and guide them through a muted paternal psychopathology that can be challenging to discern.

Finally, fathers in our sample present certain specificities. For instance, they had not experienced major changes in their professional activity nor affronted financial challenges due to COVID-19 measures, as it has been reported in literature (38, 86), thus, we may extrapolate that they were able to fully invest, focus, and explore their transition in the paternal role.

Overall, in this qualitative inquiry, fathers found to be in denial relative to this perinatal period and to the sanitary situation associated with COVID-19 and its consequences. We additionally observed the challenges of recognizing symptoms of depression and anxiety among first-time fathers, and we were surprised to assess the vulnerable state of their mental health during this period. The context of a “global pandemic embodies the overlapping of two crises, and the stress inherent to parenthood is potentially heightened by COVID-19 concerns (39, 87, p. 4).

## Limitations

Besides the novelty of this study, some methodological issues may be pointed out. Our study has focused on participants’ subjective experiences during the first antenatal period in order to understand them in a specific time and place. However, it would be interesting to explore the lived experiences from a longitudinal perspective and the perceived impact of the pandemic on these families. Moreover, our sample is quite heterogeneous in terms of paternal age, religiosity and culture (a couple were Spanish), thus these characteristics may alter their experience fatherhood. Also, we recruited fathers from a small region in France (specifically, the eastern part of the country). The two types of interviews conducted, narrative and semi-structured, lasted shorter than the usual length (48). Some fathers in our sample did not largely develop some aspects of their experience as they were sometimes unease with the silences or the prompt questions, so their responses were briefer, underlining the challenges that they may have affronted reflecting on their experience. Furthermore, they also struggled to formulate their responses during the Rorschach. Finally, the time of birth of their newborns varies: one baby was born in April, one in May, two in July, and one in August 2020. Therefore, we were unable to determine whether the different periods of COVID-19 and lockdowns are associated with the manifested signs of depression and anxiety.

## Future research and implications for practice

The results of this study shed light on the key aspects of first-time fatherhood in the unique context of the COVID-19 pandemic, which have not been explored before. This study provides valuable insights into the challenging transition to fatherhood and the limited literature available in this field.

This subject is worth further investigation as it can contribute to our understanding of men becoming fathers in the perinatal period and the psychological issues that may arise. We recommend that healthcare professionals, including family doctors, midwives, pediatricians, psychiatrists, child psychiatrists, and psychologists consider the psychological implications of this period for fathers. Analyzing the consequences of COVID-19 in the context of individual, couple, and family psychotherapy can also be beneficial.

In our study, we found that fathers experienced specific events during childbirth that caused trauma. It is important to create spaces for them to discuss their experiences, especially when they do not physically deliver themselves. This could be done through individual counseling sessions or support groups, specifically for fathers. In addition, the Rorschach test can be used therapeutically for fathers beyond its diagnostic nature.

Last but not least, we have the intention to investigate fathers in more diverse family structures as adoption, gay parenting, single dads, for example, to explore the divergences and convergences in their shared paternal experience.

## Conclusion

Our study explored the metabolizations and deferred actions, in a psychoanalytic sense, related to perinatal experiences among fathers, while also raising questions about the level of support they receive from healthcare professionals for the benefit of all three partners in the triad. It is crucial for professionals in perinatal care and early childhood to support transitions to fatherhood and to recognize the importance of the triad as a family unit. This support can help prevent and treat triadic perinatal depression, including those experienced by the mother, father, and child, rather than only focusing on postpartum depression in mothers.

## Data availability statement

The raw data supporting the conclusions of this article will be made available by the authors, without undue reservation.

## Ethics statement

Ethical approval was not required for the studies involving humans because It was a non-interventional approach. The studies were conducted in accordance with the local legislation and institutional requirements. The participants provided their written informed consent to participate in this study. Written informed consent was obtained from the individual(s) for the publication of any potentially identifiable images or data included in this article.

## Author contributions

RJ-D-P: Writing – review & editing, Writing – original draft, Visualization, Validation, Supervision, Software, Resources, Project administration, Methodology, Investigation, Funding acquisition, Formal analysis, Data curation, Conceptualization. R-AB: Writing – review & editing, Supervision, Methodology, Conceptualization. DM: Writing – review & editing, Visualization, Supervision, Formal Analysis. LR: Writing – review & editing, Visualization, Validation, Supervision, Formal analysis. CP: Writing – review & editing, Visualization, Validation, Supervision, Formal analysis. BD: Writing – review & editing, Visualization, Validation, Methodology, Formal analysis. CB: Writing – review & editing, Visualization, Validation, Supervision, Methodology, Formal analysis, Conceptualization. FK: Writing – review & editing, Writing – original draft, Visualization, Validation, Supervision, Software, Resources, Project administration, Methodology, Investigation, Funding acquisition, Formal analysis, Data curation, Conceptualization.

## Footnotes

1 These socio-professional categories are determined according to criteria of the French (INSEE or French Institute of statistics and economics studies, https://www.insee.fr/fr/statistiques/2489546#tableau-figure1_radio1).

2 During the time of the pandemic, the paternal leave lasted 14 days at that time for paternal leave. Currently, it lasts 28 days

3 Titeuf (Tootuff) is a french famous character of comic (by Zep from Switzerland).
